# Play it again, Sam: brain correlates of emotional music recognition

**DOI:** 10.3389/fpsyg.2014.00114

**Published:** 2014-02-18

**Authors:** Eckart Altenmüller, Susann Siggel, Bahram Mohammadi, Amir Samii, Thomas F. Münte

**Affiliations:** ^1^Institute of Music Physiology and Musicians's Medicine, University of Music, Drama and MediaHannover, Germany; ^2^Department of Neurology, University of LübeckLübeck, Germany; ^3^CNS Laboratory, International Neuroscience InstituteHannover, Germany

**Keywords:** musical memory, episodic memory, emotions, brain-processing

## Abstract

**Background**: Music can elicit strong emotions and can be remembered in connection with these emotions even decades later. Yet, the brain correlates of episodic memory for highly emotional music compared with less emotional music have not been examined. We therefore used fMRI to investigate brain structures activated by emotional processing of short excerpts of film music successfully retrieved from episodic long-term memory.

**Methods**: Eighteen non-musicians volunteers were exposed to 60 structurally similar pieces of film music of 10 s length with high arousal ratings and either less positive or very positive valence ratings. Two similar sets of 30 pieces were created. Each of these was presented to half of the participants during the encoding session outside of the scanner, while all stimuli were used during the second recognition session inside the MRI-scanner. During fMRI each stimulation period (10 s) was followed by a 20 s resting period during which participants pressed either the “old” or the “new” button to indicate whether they had heard the piece before.

**Results**: Musical stimuli vs. silence activated the bilateral superior temporal gyrus, right insula, right middle frontal gyrus, bilateral medial frontal gyrus and the left anterior cerebellum. Old pieces led to activation in the left medial dorsal thalamus and left midbrain compared to new pieces. For recognized vs. not recognized old pieces a focused activation in the right inferior frontal gyrus and the left cerebellum was found. Positive pieces activated the left medial frontal gyrus, the left precuneus, the right superior frontal gyrus, the left posterior cingulate, the bilateral middle temporal gyrus, and the left thalamus compared to less positive pieces.

**Conclusion**: Specific brain networks related to memory retrieval and emotional processing of symphonic film music were identified. The results imply that the valence of a music piece is important for memory performance and is recognized very fast.

## Background

Many people value music because of the emotional richness it adds to their lives (Panksepp, 1995). Music has the potential to elicit strong emotional responses, which frequently are perceived as highly pleasurable and linked to chill-sensations (for a review see Altenmüller et al., 2013). According to brain-imaging studies, such emotional arousal is linked to activation of the central nervous reward circuits and dopaminergic mechanisms, which in turn can influence cognitive abilities and memory formation (Salimpoor et al., 2011; Altenmüller and Schlaug, 2013). It therefore is not astonishing that music is often remembered and recognized for extended periods of time and linked to strong biographical memories. In the field of music psychology this phenomenon is frequently termed the “Play-it-again-Sam-Effect,” alluding to the famous movie Casablanca (Gaver and Mandler, 1987). Here, a specific song triggers strong biographical memories dating back more than a decade linked to emotions of sadness, nostalgia and remorse.

There are only a few studies investigating brain mechanisms of musical long-term memory. At present, it is still under debate, whether there is a specific memory store for music (e.g., Peretz, 1996; Ayotte et al., 2002; Peretz and Coltheart, 2003), or whether musical memories are represented in multiple stores depending on learning biography and context (Margulis et al., 2009). Recognition of familiar tunes engages the bilateral superior temporal regions and left inferior temporal and frontal areas (Ayotte et al., 2002; Plailly et al., 2007). Platel et al. (2003) have differentiated episodic and semantic musical memory. They observed frontal lobe activations in both semantic and episodic musical memory tasks. Specifically, comparison of the semantic and control tasks revealed predominately left hemispheric activation, involving the inferior frontal regions and angular gyrus in addition to bilateral medial frontal activation. In contrast, comparison of episodic and control tasks revealed predominantly right-sided activation of bilateral middle frontal regions and precuneus. Comparison of the familiar episodic and control tasks revealed activation of the right precuneus and frontal gyrus only, while comparison of the unfamiliar episodic and control tasks showed activation of the superior and middle frontal gyri and medial frontal cortex bilaterally. Thus, both familiar and unfamiliar melody recognition during the episodic task elicited frontal lobe activation, which was either right lateralized or bilateral, respectively. Interestingly, in another study from the same group, using fMRI and contrasting genuine musical and musical-semantic memory by retrieving the titles of musical excerpts, a dissociation of the genuine musical memory, mainly related to increase in BOLD response in the superior temporal lobe and musical-semantic memory, more bound to activation in the middle and lower temporal gyrus was found (Groussard et al., 2010). The situation is different, when music is linked to strong autobiographical memories. Janata (2009) assessed in an elegant paradigm the salience of autobiographical memories linked to musicals excerpts and found a clear dorsal medial prefrontal cortex activation co-varying with the degree of saliency of the memories. Three other fMRI studies have examined the neural correlates of unfamiliar music recognition. Watanabe et al. (2008) found that successful retrieval of unfamiliar musical phrases was associated with activity in the right hippocampus, the left inferior frontal gyrus, bilateral lateral temporal regions as well as the left precuneus. Plailly et al. (2007) found that unfamiliar music elicited activation of the right superior frontal gyrus and superior middle gyrus, in addition to the left central and superior precentral sulci and left parietal operculum. Finally, and in contrast to the two above mentioned studies, Klostermann et al. (2009), made a very interesting observation when presenting very short (1.8–2 s) musical clips and measuring fMRI on retrieval. They found a pronounced unilateral right posterior parietal activation related to successful retrieval of the musical clips. Furthermore, the right middle frontal gyrus contributed. Taken together, with respect to retrieval of musical memories data are contradicting.

With respect to the emotional aspects of musical appreciation, again, only a few studies have addressed this question. Very positive emotions measured as chill-intensity elicited by familiar music lead to an increase of blood flow in the left ventral striatum, the dorsomedial midbrain, the right orbitofrontal cortex, the bilateral insula, paralimbic regions, the anterior cingulate cortex, as well as the thalamus, and the bilateral cerebellum. A decrease in blood flow was found for the right amygdala, the left hippocampus, the precuneus and the ventromedial PFC (Blood and Zatorre, 2001). In another, more recent study by the same group, the neurochemical specificity of [(11)C]raclopride positron emission tomography scanning was used to assess dopamine release on the basis of the competition between endogenous dopamine and [(11)C]raclopride for binding to dopamine D_2_ receptors (Salimpoor et al., 2011). They combined dopamine-release measurements with psychophysiological measures of autonomic nervous system activity during listening to intensely pleasurable music and found endogenous dopamine release in the striatum at peak emotional arousal during music listening. To examine the time course of dopamine release, the authors used functional magnetic resonance imaging with the same stimuli and listeners, and found a functional dissociation: the caudate was more involved during the anticipation and the nucleus accumbens was more involved during the experience of peak emotional responses to music. These results indicate that intense pleasure in response to music can lead to dopamine release in the striatal system. Notably, the anticipation of an abstract reward can result in dopamine release in an anatomical pathway distinct from that associated with the peak pleasure itself. Such results may well help to explain why music is of such high value across all human societies.

Even if individuals do not have intense “chill experiences,” music can evoke activity changes in the amygdala, the ventral striatum and the hippocampus. When subjects were exposed to pleasing music, functional, and effective connectivity analyses showed that listening strongly modulated activity in a very similar network of mesolimbic structures involved in reward processing including the dopaminergic nucleus accumbens, the ventral tegmental area, the hypothalamus and insula (Menon and Levitin, 2005 review in Koelsch, 2010). Koelsch et al. (2006) compared brain responses to joyful instrumental tunes to those evoked by electronically manipulated, permanently dissonant counterparts of these tunes. During the presentation of pleasant music, increases in brain activation were observed in the ventral striatum and the anterior insula. Dissonant music, by contrast, elicited increased brain activity in the amygdala, hippocampus, and parahippocampal gyrus, regions linked to the processing of negative affect and fear. If musically untrained subjects listened to unfamiliar music that they enjoyed (Brown et al., 2004) bilateral activations in limbic and paralimbic regions were found. These were stronger in the left hemisphere, which is consistent with hypotheses about positive emotions being more strongly processed on the left (Altenmüller et al., 2002).

Memory and emotions partly share the same limbic structures, and there are strong reciprocal interactions between parahippocampal and frontal regions. The right parahippocampal gyrus is not only involved in learning and memory, but also in emotional processing of unpleasant emotions in music (Blood et al., 1999). Studies using emotional words, pictures, and events, have yielded evidence that arousal plays an important role in memory consolidation and retrieval of emotional stimuli independent of valence (Kensinger and Corkin, 2003; Phelps, 2004). Arousal facilitates focusing and directing attention to a stimulus which then is elaborated more deeply (Lane and Nadel, 2000). Interestingly, studies investigating the influence of valence on recognition and recall found a better recognition performance for either negative or positive valence compared to neutral valence independently from arousal (Anderson et al., 2003; Kensinger, 2004; Kuchinke et al., 2006). These studies pointed to a prefrontal and orbitofrontal cortex-hippocampal network to be involved in (especially positive) valence processing (Erk et al., 2005; Kuchinke et al., 2006).

In previous experiments, we found a significant valence effect when examining the retrieval of emotional vs. non-emotional music from long-term memory (Eschrich et al., 2008). Musical excerpts of symphonic film music with very positive valence attribution were better recognized than less positive pieces. Surprisingly, we could not demonstrate an arousal effect, since there was only a non-significant trend for a better recognition with increasing arousal.

To identify brain structures involved in encoding and retrieval of emotional music we conducted the present brain imaging study. We hypothesized that pieces of positive valence would be remembered better and that retrieval of these pieces would lead to activation in left prefrontal, orbitofrontal, and cingulate cortex.

To ensure strong emotional responses, highly arousing musical excerpts from symphonic film music with valence ranging from less positive (neutral) to very positive were selected.

## Methods

The local ethics committee approved the experimental protocol (Medical University Hannover) and the experiment was conducted according to the guidelines of the declaration of Helsinki.

### Participants

A group of 18 non-musicians (9 women, 2 left-handers according to Oldfield, age = 28.7 years, range = 22–49, *SD* = 8.7) gave informed consent to participate in the study for a small monetary compensation of 20 Euro. They were undergraduate and graduate students of the University of Hanover with normal hearing abilities or singers in a non-musician choir. All but three participants had learned to play an instrument or sing in a choir for at least 1–2 years to more than 10 years. Three participants had received only 1 year of musical training in primary school, learning recorder playing as foreseen in some German school curricula. The mean of musical training was 8.4 years (range = 1–15, *SD* = 5.7). Eight of the participants were still actively engaged in music making. All participants appreciated listening to music and said that music was important in their lives, in particular because of its emotional effects. Participants listened to music several times a day for altogether 1–5 h with an estimated 80% deliberately chosen. They reported to like very different types of music from folk music to classics. Most of them listened to music while doing housework or while eating and they used music to stimulate themselves or to relax. The listening situation in the laboratory was very different from their usual listening habits which might be one reason why most of them indicated that they had weaker emotional reactions to the music in the study than they usually have.

### Stimuli

Sixty excerpts of 10 s length of little known symphonic film music (mostly from so-called Hollywood B-movies) were selected from a larger pool of 160 excerpts on the basis of valence and arousal rating results. We included stimuli of an earlier study (Eschrich et al., 2008) and added new excerpts, which were selected in a further rating study performed with 37 participants (14 men, mean age = 28.6, range = 19–49 years). This selection process identified 60 structurally similar pieces with identical high arousal ratings but varying valence ratings. The loudness of all musical excerpts was normalized. We calculated the power spectrum for each piece per channel (Hanning window: sample rate = 44100 Hz; FFT size = 16384; maximum frequency resolution = 2.692 Hz) resulting in the relative amplitude for every frequency per channel. After this normalization procedure, the amplitude peaks per frequency band did not show differences between positive and less positive valence pieces (Mann-Whitney U-Test).

Two sets of 30 pieces were created with a comparable distribution of emotional and structural features. Each of these was presented to half of the participants during the first session outside of the scanner (encoding phase), while all stimuli were used during the second session (recognition phase) which took place inside the MRI-scanner.

After the experiment, the two sets of items were compared according to the participants' ratings of arousal and valence as well as recognition performance. Both item sets did not differ significantly with respect to any of these variables (Mann-Whitney-U-test for arousal, *p* = 0.9; valence, *p* = 0.17). Recognition performance of the participants did not differ between item sets (*d*′, *p* = 0.44).

### Questionnaires

We used self-developed questionnaires based on bipolar five-point rating-scales. After each piece of music, arousal, valence, and emotional intensity had to be rated on a five-point rating-scale (arousal: 1 = very relaxing/calming to 5 = very arousing; valence: 1 = less positive to 5 = very positive; for emotions felt and emotions perceived separately). We used “less positive” (in German: wenig positiv) instead of “negative,” because in a pretest none of the music pieces received a “negative” rating. In a mood questionnaire participants were asked to rate their present state of arousal and valence at the beginning of each session. At the end of the first session participants filled out a questionnaire regarding demographic data, their musical knowledge, expertise, listening attitudes as well as music preferences and experience (expertise questionnaire).

### Procedure

During the first experimental session, participants sat in a comfortable chair with a computer keyboard on their knees, and listened to the stimuli via closed headphones (Beyerdynamic DT 770 PRO) and an USB soundcard (Audiophile, M-Audio). Questions and answer options appeared on the computer screen. Answers were logged by keyboard presses. In both sessions, prior to the music rating, participants filled out a short mood questionnaire. After this participants received written and oral instructions for the experiments. Prior to the experiment proper, three practice excerpts were given. During each trial, participants listened to the excerpt of a musical work of 10 s of length. Subsequently, participants pressed a button to start the valence, arousal, and liking rating questions on the screen. Responses were not timed. After the last question there was a break of 10 s, before the new excerpt started. Excerpts were presented in randomized order in two blocks of 15 pieces, which were separated by short breaks. The experiment was run using “Presentation.”

During the encoding phase, participants were unaware of the subsequent recognition task in the second session. At the end of the first session participants filled out the expertise questionnaire. In the second session, on the next day, participants lay in the scanner and listened to the 30 old stimuli from the last session randomly inter-mixed with 30 new pieces. All participants had to make an old/new decision after each piece by pressing one of two buttons.

### Data analysis

Musical excerpts were categorized according to the pre-defined valence categories (less positive and very positive). For each participant *d*′ was computed for the entire set of stimuli and separately for each valence category. The *d*′ values per category were compared using Friedman tests and a Dunn's multiple comparison test as *post-hoc* test. For the analysis of the influence of musical structural features on the “recognizability” of the pieces a regression tree analysis was used (Cart 6.0, Salford Systems, default adjustments). As dependent variable *d*′ was calculated per musical piece rather than per participant to serve as a measure for recognizability of a certain piece of music. For half of the participants a certain piece of music was a target piece (hits), for the other half of participants it was a distractor piece (false alarms). Thus, the recognizability measure was based on empirical data from the experiment. The least square method was used to find the optimal tree.

### fMRI procedure

A slow event related design was used for the stimulus presentation. Each stimulation period (10 s) was followed by a 20 s resting period during which participants pressed the answer button (one button for “old,” the other for “new”).

Stimuli were presented via fMRI compatible electrodynamic headphones integrated into earmuffs for reduction of residual background scanner noise (Baumgart et al., 1998). The sound level of stimuli was individually adjusted to good audibility.

### Image acquisition

Magnetic-resonance images were acquired on a 3T Allegra Siemens Scanner equipped with a standard 8-channel head coil. A total of 650 T^*^_2_-weighted volumes of the whole brain (*TR* = 2000 ms, *TE* = 30 ms, flip angle = 80°, FOV = 224 mm, matrix = 64^2^, 30 slices, slice thickness = 3.5 mm, interslice gap = 0.35 mm, one run of 907 volumes) near to standard bicommisural (ACPC) orientation were collected. After the functional measurement T_1_-weighted images (*TR* = 1550 ms, *TE* = 7.3 ms, flip angle = 70°, FOV = 224 mm, and matrix = 256^2^) with slice orientation identical to the functional measurement were acquired to serve as a structural overlay. Additionally, a 3D high resolution T_1_-weighted volume for cortex surface reconstruction (FLASH, *TR* = 15 ms, *TE* = 4.9 ms, flip angle = 25°, matrix = 1.2 × 256^2^, 1 mm isovoxel) was recorded. The participant's head was fixed during the entire measurement to avoid head movements.

### fMRI data analysis

First the participant's head motion was detected by using Brain Voyager QX software. All datasets were motion- and slice scan time corrected prior to further analysis. Additional linear trends and non-linear drifts were removed by temporal filtering using a high-pass filter of 128 s. Finally, after the co-registration with the structural data, a spatial transformation into the standard Talairach space (Talairach and Tournoux, 1988) was performed.

To identify possible regions of activity group data were analyzed by multi-subject GLM in standard space. To emphasize spatially coherent activation patterns, functional data was additional spatially smoothed with a Gaussian kernel of 8 mm full width at half maximum.

Five different GLM were defined: The first compared the stimuli with silence. The second one compared less positive and positive pieces. The third GLM contrasted old (target) with new (distractor) pieces. The fourth one compared recognized with not recognized targets while the last GLM compared recognized positive with recognized less positive targets. These GLMs were used to disentangle memory retrieval effects as well as valence effects on recognition: Contrasting neutral and positive valence (over all pieces and only for recognized targets) trials yields valence related activations. The contrast of old and new stimuli as well as recognized and not recognized targets reflects memory (retrieval) effects. Statistical maps were created using a threshold of *p* < 0.001. When using a FEW-correction for multiple comparisons, statistical results did not reveal significant differences, therefore we used uncorrected for multiple comparisons. As significant results, we applied *p* < 0.05 (Bonferroni corrected for the comparison between silence and music) with a cluster threshold of 20 voxels. We decided to provide the results of several GLMs instead which comprise all contrasts that would have been examined in decomposing a Two-Way ANOVA and, in addition, a number of other contrast. Please note, that given the fact that in some contrasts results did not survive rigorous correction procedure we decided to provide SPMs at less strict thresholds to allow descriptive data analysis in the sense of Abt (1987).

## Results

### Overall recognition performance and valence effect

The number of correctly recognized targets (*n* = 30) differed among participants from 9 to 22 with a median of 15. The *d*′ values ranged from −0.52 to 1.59 with a mean of 0.25. One participant with the very low *d*′ had a hit ratio on chance level (0.5) but a very high false alarms ratio (0.7). It was verified that he had not mistaken the assignment of the keys.

No significant effect of valence on recognition performance was found (less positive *d*′ = 0.34; very positive *d*′ = 0.16). A floor effect might have prevented the detection of a valence effect on recognition because of the low overall recognition rate.

Participants' ratings in the first session confirmed that the pieces were perceived as arousing (eight pieces with a median of 2, 52 ratings had a median of three or above) and either less positive (26 of 30 pieces were rated as such) or very positive (29 or 30).

The selected music pieces were indeed unfamiliar to the participants, with one participant knowing 3, one participant knowing 2, and two participants knowing 1 piece from prior exposure. No piece was known by more than one participant. Even if a participant had indicated to know a piece during encoding, he/she did not necessarily recognize this piece in the recognition session. We therefore decided to include all pieces of music in the analysis.

### Imaging data

**Figure 1:**
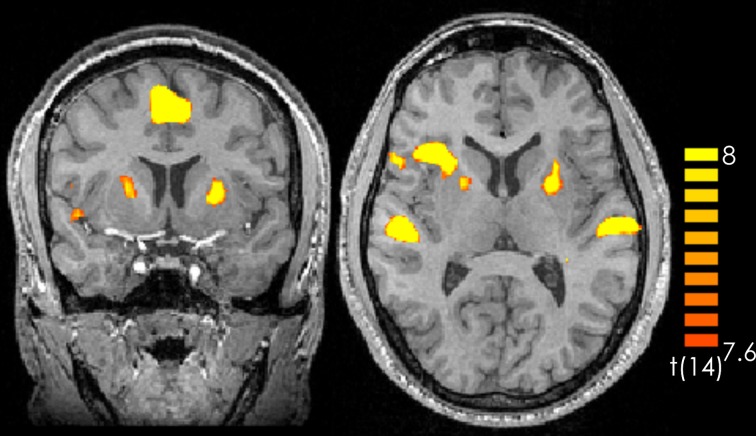
**Contrast of music > silence over all participants and all music pieces.** The yellow-colored regions represent activation during the music presentation. *p* < 0.05 (Bonferroni corrected).

Comparing silence with music (music > silence) yielded activation in the right and left superior temporal gyrus, the right insula, the right middle frontal gyrus as well as the bilateral medial frontal gyrus and the left anterior cerebellum (Figure 1 and Table 1). These results confirm previous experiments showing the important role of the superior temporal gyrus, the middle frontal gyrus and the insula in hearing in general as well as music perception and detection (for a review see Peretz and Zatorre, 2005). There were no significant activations for silence > music.

**Table 1:** **Laterality (R, right, L, left), coordinates and *t*-values for every contrast and active brain region**.

**Region of activation**	**Laterality**	**Coordinates**	***t*-value**
**STIMULI > SILENCE**			
Medial frontal gyrus	R/L	0, 2, 55	12.45
Middle frontal gyrus	R	38, 3, 40	9.322
Insula	R	34, 18, 10	9.93
Superior temporal gyrus	R	49, −19, 10	9.46
Superior temporal gyrus	L	53, −17, 10	9.70
Anterior lobe, Cerebellum (Culmen)	L	−28, −50, −26	8.75
**OLD > NEW PIECES**			
Thalamus (Medial dorsal nuclei)	L	−7, −12, 10	4.27
Midbrain	L	−2, −23, −8	4.34
**NEW > OLD PIECES**			
Middle frontal gyrus	R	41, 58, 10	−4.40
**RECOGNIZED > NOT RECOGNIZED TARGET PIECES**			
Inferior frontal gyrus	R	27, 19, −17	4.3
Cerebellum	L	−27, −53, −32	4.27
**POSITIVE PIECES > LESS POSITIVE PIECES**			
Medial frontal gyrus	L	−1, 39, 31	4.52
Posterior cingulate cortex	L	0, −34, 31	4.32
Precuneus	L	−6, −62, 31	4.34
Superior frontal gyrus	R	29, 43, 14	4.66
Thalamus	L	−18, −12, 13	4.39
Middle temporal gyrus	L	−59, −36, 1	4.52
Middle temporal gyrus	R	56, −11, −14	4.82
Cerebellum, posterior	L	−3, −50, −36	4.28
Cerebellum, posterior	L	33, −57, −38	4.40
**RECOGNIZED POSITIVE > RECOGNIZED LESS POSITIVE TARGET PIECES**			
Middle frontal gyrus	L	−25, 10, 43	4.34
Precentral gyrus	L	−46, 4, 34	4.18
Posterior cingulate gyrus	L	−4, −26, 29	4.14
Precuneus	L	−13, −57, 23	4.31
Medial frontal gyrus	R/L	7, 53, 16	4.40
Superior frontal Gyrus	L	−53, −41, 16	4.30
Thalamus	L	−18, −11, 13	4.26
Posterior cingulate gyrus	L	−10, −54, 10	4.13
Thalamus	L	−3, −7, 9	4.17
Thalamus	R	21, −19, 1	4.19
Cerebellum, anterior lobe	R/L	0, −52, −2	4.28
Superior temporal gyrus	R	51, 13, −2	4.93
Superior temporal gyrus	R	56, −10, −2	4.15
Cerebellum, posterior lobe	R	20, −62, −35	4.44

The table shows only the significant contrasts (Stimuli > silence: p < 0.05, Bonferroni corrected; for the other contrasts: p < 0.001, not corrected).

For the contrast old > new pieces activation in the medial dorsal nucleus of the left thalamus and in the left midbrain was found. Interestingly the reverse contrast (new > old) yielded activation in the right middle frontal gyrus (Table 1).

**Figure 2:**
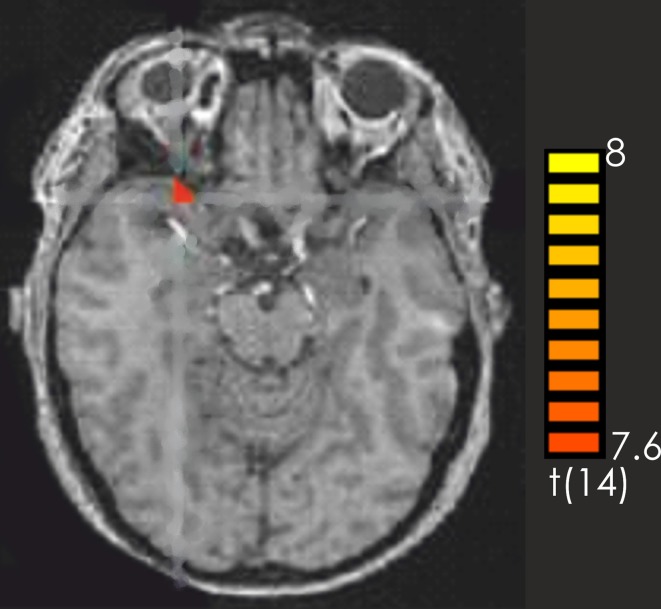
**Contrast of recognized > not recognized target pieces over all participants.** The red-colored regions represent activation for the recognized target pieces. *p* < 0.001 (not corrected).

Consistent with the findings for the old vs. new contrast there was a focused activation in the right inferior frontal gyrus and the left cerebellum for recognized vs. not recognized targets (Figure 2). For the reverse contrast no activations were seen (Table 1).

The contrast positive > less positive pieces yielded predominantly left-lateralized activations, in particular in the left medial frontal gyrus, the left precuneus, the left posterior cingulate, the left thalamus as well as the bilateral middle temporal gyrus, and the right superior frontal gyrus. There was also activation in the posterior cerebellum bilaterally. No activations were discovered for the contrast less positive > positive (Table 1).

**Figure 3:**
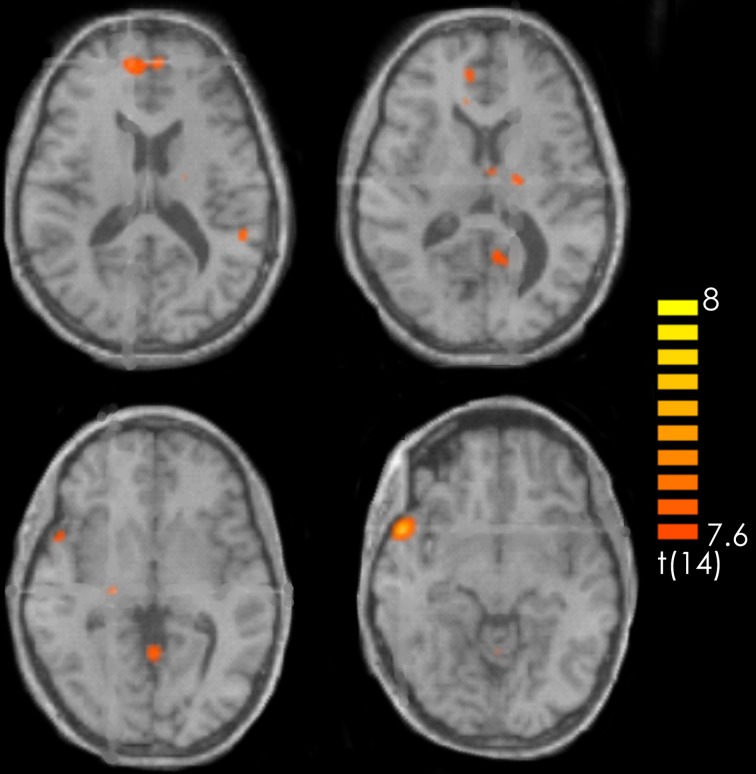
**Contrast of recognized positive > recognized less positive target pieces over all participants.** The red-colored regions represent activation for the recognized positive target pieces. *p* < 0.001 (not corrected).

The contrast recognized positive > recognized less positive yielded activation in the left superior and middle frontal gyrus, the bilateral medial frontal gyrus, the right superior temporal gyrus and the temporal pole, the left posterior cingulate and the left precuneus. Furthermore, activations were observed in the left precentral gyrus, the bilateral thalamus as well as in the bilateral anterior cerebellum and the right posterior cerebellum (Figure 3, Table 1).

## Discussion

This study addressed the neural basis of emotional musical long-term memory by means of fMRI in a recognition task.

Surprisingly, and in contrast to our previous study (Eschrich et al., 2008), we could not replicate the valence effect. Participants did not remember those musical excerpts better, which they had rated emotionally highly positive in the encoding phase. As the overall recognition performance was quite low, this may reflect a floor effect. It was rather difficult to find suitable stimuli for the recognition task which were structurally similar as to avoid that structural features of the music would have a bigger influence on recognition and fMRI activations than the emotional component. Yet, pieces had to differ in emotional effect and be different enough to be recognized. Due to constraints in time that can be spent in the scanner, music excerpts were rather short (10 s) which could have further contributed to recognition problems. As stimuli varied only on the valence dimension with arousal on a high level for all pieces, it might have been difficult for the participants to differentiate between the pieces and to feel a clear emotional difference. Thus, although we had conducted an extensive rating study, the stimulus selection might not have been optimal. Additionally, scanner-noise during the retrieval might have interfered with both, recognition and emotion induction. It can be excluded that subjects suffering from an amusic disorder participated in the study, since we included only subjects who reported interest in music and we even assessed daily time of listening to music, which ranged between 0.5 and 5 h.

The low recognition rate might also explain why we only found thalamic and midbrain activity for the comparison of old to new music pieces and only activation in the right inferior frontal gyrus for the contrast of recognized vs. not recognized pieces. Retrieval processes from long-term representations of music tend to engage inferior frontal regions (Zatorre et al., 1996; Halpern and Zatorre, 1999; Zatorre and Halpern, 2005). Also studies in other domains show the importance of these brain regions for memory retrieval in general (e.g., Nyberg et al., 1996). Among the many functions assigned to the inferior frontal gyrus have been working memory (Zatorre et al., 1994; Holcomb et al., 1998), and the perceptual analysis of melodies (Fletcher and Henson, 2001). In particular, dorsolateral and inferior frontal areas are most often recruited when working memory load is high (Zatorre et al., 1994; Griffiths et al., 1999). However, according to other studies activation for musical memory retrieval would have been expected in inferior frontal and temporal regions as well as the superior temporal gyrus (Halpern and Zatorre, 1999; Platel et al., 2003; Rugg et al., 2003; Peretz and Zatorre, 2005). The activation in the left cerebellum might be due to hand motor control as the participants answered by button presses of the right hand (Platel et al., 2003). Our data indicate an involvement of the inferior frontal gyrus in the retrieval from musical long-term memory. However, further experiments examining the brain regions responsible for musical long-term memory are needed.

The hypothesized valence effect was confirmed concerning the left-lateralization of activation for the very positive stimuli and activity in frontal brain regions. The mainly left-sided activation of the frontal and temporal gyrus as well as the cingulate cortex confirm the role of these structures for emotion processing and corroborate earlier studies (Altenmüller et al., 2002; Davidson, 2003). The precuneus has been implicated in memory-related and selective attention processes and does not seem to be specific for emotions (Berthoz, 1994). Bilateral activity of the cerebellum when listening to emotional music has also been found in other studies, (e.g., Blood and Zatorre, 2001) although it has to be acknowledged that its role is not well understood (Koelsch, 2010). There was no specific activity for the contrast of less positive > very positive stimuli which might indicate that participants perceived the less positive pieces as emotionally neutral rather than negative, and thus none of the brain regions typically associated with negative affect were activated.

Concerning the contrast of recognized positive > recognized less positive stimuli the role of the posterior cingulate gyrus in emotion control (Blood et al., 1999; Koelsch et al., 2005, 2006; Ochsner and Gross, 2005; Masaki et al., 2006) as well as the role of frontal regions in the processing of complex stimuli and their valence (Kensinger and Corkin, 2003; Kensinger, 2004) was mostly confirmed. The cingulate gyrus seems also to be involved in episodic memory processing (Critchley, 2005). The right temporal pole was found to be active in the processing of positively valenced stimuli (e.g., Piefke et al., 2003; Brown et al., 2004; Ethofer et al., 2006; Jatzko et al., 2006). Thus, the respective regions most probably are involved in emotion (positive valence) processing. However, it should also be mentioned that there are data, not fitting into this scheme. In the study by Klostermann et al. (2009) right parietal and right middle frontal areas were related to memory retrieval and degree of pleasantness. Possibly this somehow isolated result can be ascribed to the different nature of the stimuli, which were extremely short and were explicitly composed containing novel sounds and timbres.

Surprisingly, and in contrast to many studies on brain correlates of emotional processing of music (Koelsch, 2010), no activation in the orbitofrontal and prefrontal cortex was found. We can only speculate about the reasons: The emotion variation in the different categories might not have been salient enough. Also, the short duration of the music pieces (10 s) and the presentation in the scanner might have precluded emotion induction.

As the right superior temporal gyrus and the middle and superior frontal gyrus were active for the contrast “recognized positive > recognized less positive” but not in the “positive > less positive” comparison, these regions seem to be involved in the recognition of emotional music.

This fMRI study was a first exploratory attempt to identify the neural underpinnings of emotional musical memory. Further experiments will be needed to clarify this issue in more detail. An idea to make emotional information more salient would be to compare music pieces with low arousal and less positive valence with pieces with high arousal and very positive valence, although in this case it would not be possible to disentangle the influence of arousal and valence on recognition performance and on brain activity. Additionally a sparse temporal sampling design (cf. Szycik et al., 2008) to avoid the loud scanner noise and make the situation more appropriate for appreciating and recognizing the music.

### Conflict of interest statement

The authors declare that the research was conducted in the absence of any commercial or financial relationships that could be construed as a potential conflict of interest.
